# White Matter Damage in the Cholinergic System Contributes to Cognitive Impairment in Subcortical Vascular Cognitive Impairment, No Dementia

**DOI:** 10.3389/fnagi.2017.00047

**Published:** 2017-02-27

**Authors:** Qing Liu, Zude Zhu, Stefan J. Teipel, Jianwei Yang, Yi Xing, Yi Tang, Jianping Jia

**Affiliations:** ^1^Department of Neurology, Xuan Wu Hospital, Capital Medical UniversityBeijing, China; ^2^Collaborative Innovation Center for Language Competence, Jiangsu Normal UniversityXuzhou, China; ^3^Department of Psychosomatic Medicine, University of RostockRostock, Germany; ^4^German Center for Neurodegenerative DisordersRostock, Germany; ^5^Department of Neurology, Beijing Friendship Hospital, Capital Medical UniversityBeijing, China; ^6^Center of Alzheimer’s Disease, Beijing Institute for Brain Disorders; Beijing Key Laboratory of Geriatric Cognitive Disorders; Neurodegenerative Laboratory of Ministry of Education of the People’s Republic of ChinaBeijing, China

**Keywords:** vascular cognitive impairment no dementia, cholinergic system, magnetic resonance imaging, tractography, cognitive impairment

## Abstract

Cholinergic deficiency has been implicated in the pathogenesis of vascular cognitive impairment (VCI), but the extent of involvement and underlying mechanism remain unclear. In this study, targeting the early stage of VCI, we determined regional atrophy within the basal forebrain and deficiency in cholinergic pathways in 25 patients with vascular cognitive impairment no dementia (VCIND) compared to 24 healthy elderly subjects. By applying stereotaxic cytoarchitectonic maps of the nucleus basalis of Meynert (NbM), no significant atrophy was identified in VCIND. Using probabilistic tractography analysis, our study tracked the two major white matter tracks which map to cholinergic pathways. We identified significantly lower fractional anisotropy (FA) in VCIND. Mediation analysis demonstrated that FA in the tracked pathways could fully account for the executive dysfunction, and partly mediate the memory and global cognition impairment. Our study suggests that the fibers mapped to the cholinergic pathways, but not the NbM, are significantly impaired in VCIND. MRI-based *in vivo* tracking of cholinergic pathways together with NbM measurement may become a valuable *in vivo* marker for evaluating the cholinergic system in cognitive disorders.

## Introduction

The precise mechanisms underlying vascular cognitive impairment (VCI), the second common cause of dementia (Kalaria et al., 2008), remain unclear. Several lines of evidence suggest a central role for cholinergic deficiency. First, both cholinergic neuronal deficits (Keverne et al., 2007) and cholinergic denervation (Mesulam et al., 2003) have been identified in patients with cerebral autosomal dominant arteriopathy with subcortical infarcts and leukoencephalopathy (CADASIL), a purely genetic form of vascular dementia (VaD). Secondly, significantly decreased cerebrospinal fluid acetylcholine concentrations were observed in patients with Binswanger or multi-infarct dementia (Tohgi et al., 1996; Wallin et al., 2003). Finally, clinical trials have prompted potential beneficial effects of cholinergic drugs in patients with VaD (Erkinjuntti et al., 2002; Black et al., 2003; Wilkinson et al., 2003).

However, the exact nature of cholinergic involvement in VCI is still unresolved. Postmortem studies in patients with Binswanger type VaD (Tomimoto et al., 2005) and subcortical VaD (Jung et al., 2012) have revealed relative preservation of the nucleus basalis of Meynert (NbM) neurons with impaired subinsular cholinergic fibers. An *in vivo* study showed NbM atrophy in patients with subcortical ischemic VaD (Kim et al., 2013). One possible explanation for this discrepancy may be methodological limitations, e.g., the inability to obtain accurate *in vivo* measurements of the NbM. The substantia innominata (SI) thickness has been used to assess NbM atrophy (Hanyu et al., 2007; Kim et al., 2013). Nevertheless, 2D measurements of SI thickness have only provided a very rough estimate of NbM volume (Teipel et al., 2005). Using the recently developed stereotaxic cytoarchitectonic maps of the NbM based on combined magnetic resonance imaging and histology of postmortem brains, volume changes in NbM have been more accurately measured (Teipel et al., 2005; Grothe et al., 2010). Moreover, as Alzheimer’s disease (AD) research has already addressed the importance of targeting early stages of the disease process (Sperling et al., 2014), it is important to further evaluate cholinergic involvement during early stages of VCI, i.e., vascular cognitive impairment, no dementia (VCIND) as well.

In addition to the volume change in the NbM region, studies have tried to investigate the white matter (WM) microstructure of the cholinergic pathways (Behl et al., 2007; Kim et al., 2013). The cholinergic input that innervate the cerebral cortex mostly originate from the NbM Ch4 region (Mesulam and Geula, 1988), and their spatial distribution has been identified, following medial and lateral cholinergic pathways that were observed in a postmortem study (Selden et al., 1998). Previous studies have visually rated WM hyperintensities within cholinergic pathways using the cholinergic pathways hyperintensities scale (CHIPS) (Behl et al., 2007; Kim et al., 2013). However, this approach did not reflect the spatial damage of the whole cholinergic network or reveal the underlying mechanism. Diffusion tensor imaging (DTI) is an *in vivo* technology (Beaulieu, 2002) that is used to investigate the WM microstructural integrity. Probabilistic tractography can be applied to specifically investigate the WM tracks that are mapping to the cholinergic pathways by tracking fiber connections between regions of interest (ROIs) such as the NbM and the cingulum. However, so far, only the medial pathway was tracked in cognitively healthy participants (Hong and Jang, 2010), leaving it an open question whether the lateral pathway can also be tracked.

Our study aimed to investigate the role of cholinergic system changes in the pathogenesis of VCI. In pursuit of this aim, we focused on patients with subcortical VCIND, which is a relatively homogeneous condition and is supposed to reflect early pathophysiological changes in VCI. Using probabilistic tractography analysis, we tracked the fibers along with the two major cholinergic pathways. In combination with stereotaxic cytoarchitectonic maps of the NbM, we investigated the contribution of the damage in the cholinergic system to cognitive impairment in VCIND.

## Materials and Methods

### Subjects

Twenty-five consecutive patients with subcortical VCIND (47–69 years old) were prospectively recruited from the memory clinic at Xuan Wu Hospital, Capital Medical University. All patients were diagnosed by a consensus panel including three senior neurologists and met the following inclusion criteria: (1) Literate Han Chinese with a consistent caregiver (>4 days/week); (2) complaint and/or informant report of cognitive impairment involving memory and/or other cognitive domains lasting for at least 3 months; (3) neither normal nor demented according to the criteria of the Diagnostic and Statistical Manual of Mental Disorders, Fourth Edition (Winblad et al., 2004), comprising the following criteria: a clinical dementia rating (CDR) ≥0.5 on at least one domain (Hughes et al., 1982) and global score = 0.5, and a Mini-Mental State Examination (MMSE) score ≥ 20 (primary school) or ≥24 (junior school or above) (Folstein et al., 1975; Zhang et al., 1990); and (4) normal or slightly impaired daily living activities as defined by a total score of ≤1.5 for the three functional CDR domains (home and hobbies, community affairs, and personal care) (Zhang et al., 1990). Participants were excluded who had any conditions that would preclude completion of neuropsychological testing or disorders other than subcortical VCIND that would affect cognition.

All patients meeting the clinical criteria underwent brain MRI including hippocampal assessment at screening. The MRI-based entry criteria details were as follows: (1) multiple (≥3) supratentorial subcortical small infarcts (3–20 mm in diameter) with/without white matter lesions (WML) of any degree or moderate to severe WML (score ≥ 2 according to the Fazekas rating scale; Fazekas et al., 1987) with/without small infarct; (2) absence of cortical or watershed infarcts, hemorrhages, hydrocephalus, or WML with specific causes (e.g., multiple sclerosis); and (3) no hippocampal or entorhinal cortex atrophy (score 0 according to the medial temporal lobe atrophy scale of Scheltens; Scheltens et al., 1992).

Twenty-four healthy elderly subjects, free from a history of any major medical, neurological, or psychiatric illness, were recruited from the community and served as a control group. No apparent abnormal findings or cognitive impairments were expected to be identified in MRI or neuropsychological testing.

This study received approval from the Xuan Wu Hospital institutional review board and the methods were carried out in accordance with the Declaration of Helsinki. Informed written consent was obtained from each participant.

### Neuropsychological Assessment

Neuropsychological evaluations included the MMSE, the Montreal Cognitive Assessment (MoCA), the CDR scales, Digit Span, the Trail Making Test (TMT) A and B, the WHO-UCLA Auditory Verbal Learning Test (AVLT), the Boston Naming Test (BNT), the Hachinski Ischemic Scale (HIS), the Geriatric Depression Scale (GDS), the Neuropsychiatric Inventory (NPI) and the Activities of Daily Living (ADL) assessment.

### Imaging Data Acquisition

Imaging data were collected on a 3 Tesla Siemens scanner. T1 images with high spatial resolution, DTI images and Flair images were collected. Brain structure data were collected with a three-dimensional (3D) magnetization-prepared rapid gradient echo (MP-RAGE) scan [repetition time (TR) = 1690 ms, echo time (TE) = 2.56 ms, flip angle = 12°, 1-mm isotropic voxels covering the whole brain]. Diffusion data were collected using a double spin-echo EPI sequence (TR = 8000 ms, TE = 96 ms, flip angle = 90°, FOV = 224 mm^2^, in-plane resolution = 1.75 × 1.75 mm^2^ voxels, 54 contiguous 2-mm-thick axial slices) with 64 non-collinear encoding directions (*b* = 1000 s/mm^2^) and 11 images without diffusion weighting (*b* = 0 s/mm^2^, b0). Flair images were acquired for Fazekas rating (TR = 8000 ms, TE = 140 ms, time inverse = 2000 ms, FOC = 224 mm, thickness = 6 mm, 1 mm gap, 20 slices). The DTI images were collected with alignment to the AC-PC line, and part of the cerebellum was missed for some subjects to include the cerebral cortex.

### Voxel-Based Morphometric Analysis

Analysis was conducted blind to diagnosis. To extract the NbM volume, each participant’s T1 image was used to perform voxel-based morphometric (VBM) analysis following the procedure in FSL-VBM. The structural images were brain-extracted and gray matter-segmented before being registered to the MNI 152 standard space using non-linear registration. The resulting images were averaged and flipped along the *x*-axis to create a left–right symmetric, study-specific gray matter template. Second, all native gray matter images were non-linearly registered to this study-specific template and “modulated” to correct for local expansion (or contraction) due to the non-linear component of the spatial transformation. The modulated gray matter images were then smoothed with an isotropic Gaussian kernel with a sigma of 3 mm. After preprocessing, the NbM template, which was adopted from a previous study (Kilimann et al., 2014), was registered to native T1 space to extract the gray matter volume for each participant.

### Probabilistic Tractography

**FIGURE 1:**
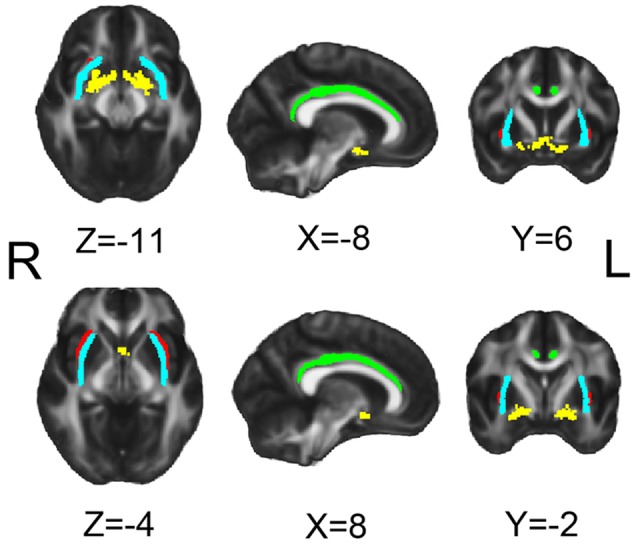
**Seeds for white matter tracking.** Yellow for NbM, green for bilateral cingulum (Cing), light blue for external capsule (ExCap) and red for claustrum (Claus).

The DTI data were analyzed using the FSL toolbox. In the tractography analysis, the four ROIs which are illustrated in the MNI space in **Figure 1** were NbM, bilateral cingulum (Cing), bilateral external capsule (ExCap), and bilateral claustrum (Claus). The NbM template was adopted from a previous study (Kilimann et al., 2014). The Cing and ExCap ROIs were selected from the International Consortium of Brain Mapping-DTI WM labels atlas. The Claus ROIs were defined in each participant’s native TI space. Probabilistic tracking in native DTI space was performed following procedures recommended by FSL with a 2-ROI approach, as described in detail in our previous work (Feng et al., 2016). Specifically, ROIs except for Claus were coregistered to the participant’s native DTI space before tractography analysis. For the Cing and ExCap ROIs, the registration parameters of individual DTI image to standard DTI image were inversely applied, to register the Cing and ExCap from standard space to native space. As the NbM ROI was originally in MNI space, the registration parameters from standard diffusion space to standard T1 MNI space was inversely applied to the NbM ROI, then was registered to native diffusion space. DTI raw images were preprocessed, including correction for motion and residual eddy current with the non-brain tissues being removed. DTI fit was applied before the algorithm implemented in FSL (BedpostX) was used to calculate the diffusion parameters for each voxel. Probabilistic tracking was then performed by repeating 5,000 random samples from the NbM seed ROI voxels to the second ROI (Cing, Claus or ExCap) voxels in a 2-ROI approach. These streamline samples started at the first ROI voxels and propagated through the local probability density functions of the estimated diffusion parameters. When a 2-ROI approach was used, only those streamlines initiated from the first ROI that reached a voxel in the second ROI (or vice versa) were retained. For each pathway, a waytotal-normalized proportion map was used to generate a group mask with a threshold at 80% of successful streamlines passing through a given voxel at the group level in each VCIND and Control group. The group mask was restricted to those voxels within the mean fractional anisotropy (FA) skeleton to limit the effects of partial voluming.

### Diffusivity Indices Extraction

To better character the microstructure change in the cholinergic pathway, diffusivity indices were extracted for analysis. FA is an integrated measurement for WM microstructure. FA can be decomposed into three mutually orthogonal eigenvectors to reflect the diffusion coefficient along the direction of “apparent diffusion” (axial diffusivity, DA), and the diffusion coefficients along two orthogonal directions embedded in the plane perpendicular to the main diffusion direction. The averaged of the two latter orthogonal eigenvectors is radial diffusivity (DR). The sum of the three eigenvectors is mean diffusivity (MD). Mode of anisotropy represents the shape of the fiber. Larger mode of anisotropy (MO) was associated with more linear shape. According to animal and human studies, different diffusivity alteration patterns were associated with varied mechanisms (Madden et al., 2012). To extract these diffusivity indices, participants’ DTI datasets were normalized to MNI152 (1 mm × 1 mm × 1 mm) space using FSL to enable selection of spatially corresponding cholinergic pathway ROIs across subjects (described below). Registration of FA images into MNI space followed a series of procedures known as Tract-Based Spatial Statistics (TBSS) (Smith et al., 2006). The raw images were corrected for motion and residual eddy current with the non-brain tissues being removed. The eddy current corrected FA datasets were then affinely registered and resampled to the 1-mm isotropic MNI152 space. All MNI-transformed FA images were then averaged to generate a mean FA image that was used to create a common WM tract skeleton. This skeleton was then thresholded at an FA value of 0.2 to minimize partial volume effects after warping across subjects. Each participant’s aligned FA image was subsequently projected onto the FA skeleton to account for residual misalignments between participants after the initial non-linear registration. Following the non-FA procedure provided by FSL, MD, axial diffusivity (DA), radial diffusivity (DR) and mode of anisotropy (MO) were also projected onto the skeleton for each participant. Mean FA, MD, DA, DR and MO values were then extracted from the skeletonized cholinergic pathway masks for each participant.

### Statistical Analysis

Demographics were compared between groups using independent *t*-tests for age and education and Chi-square tests for gender. As the CDR rating was 0.5 for each VCIND participant and 0 for control participants, we used the CDR sum of boxes for group comparisons in a one-way analysis of variance with covariates (ANCOVA), controlling for age, gender and education. A similar procedure was conducted for each neuropsychological test.

After intracranial volume correction, the NbM volume was compared between groups with one-way ANOVA and ANCOVA, with and without controlling for age, gender and education. The same ANCOVA procedure was applied for testing the group difference in diffusivity indices.

To test whether brain structure contributed to neuropsychological scores across groups, we performed a partial correlation between neuropsychological scores, FA and volume values, controlling for demographic difference, i.e., age, sex, and education. CDR related correlation was performed in the VCIND group only.

The mediation analyses sought to determine whether the observed relationships between group and neuropsychological scores could be better accounted for (i.e., mediated) by WM microstructure (i.e., FA), following a widely used procedure suggested by Salthouse (1993). To limit the statistical comparisons, the FA, which integrates multiple diffusivity components, was used in the mediation analysis. We followed Baron and Kenny’s criterion for mediation analysis (Baron and Kenny, 1986), which requires that all three variables entered into a model be reliably correlated. To examine whether WM microstructure mediated the relationship between group and neuropsychological scores, we used hierarchical regression analyses in which group was entered as a predictor of neuropsychological scores both alone and after entering WM microstructure into the model. Finally, we calculated the degree to which each mediator attenuated the amount of variance in neuropsychological scores that can be explained by group, following a widely used procedure suggested by Salthouse (1993).

## Results

### Demographics

Demographic findings are shown in **Table 1**. The patients and controls showed significant differences in gender, but comparable age and years of education. As indicated by the HIS and Fazekas scores, the VCIND group exhibited significantly higher vascular factors and WM lesions. The Control group outperformed the VCIND group in each of the neuropsychological tests. For CDR ratings, all of the VCIND patients were rated at 0.5 and all of the controls were rated at 0. The CDR sum of boxes (CDR-SB) showed variability across VCIND participants and was used for subsequent analyses.

**Table 1:** Characteristics of controls and patients.

	Control	VCIND	*t / x^2^/ F*
**Demographic characteristics**			
*N*	24	25	
Age	59.4 (6.3)	60.8 (5.9)	0.8
Education	11.4 (2.8)	11.3 (2.9)	0.1
Gender (M/F)	4/20	14/11	8.2ˆ**
**Clinical characteristics**			
HIS	0.2 (0.4)	4.6 (3.7)	28.6ˆ***
Fazekas	0	3.3 (1.1)	159.8ˆ***
**Neuropsychological scores**			
MMSE	29.0 (1.1)	27.1 (1.8)	16.3ˆ***
MoCA	27.0 (1.8)	23.0 (3.2)	25.5ˆ***
CDT	14.1 (0.8)	13.3 (1.3)	4.2ˆ*
ADL	20.0 (0)	21.4 (3.2)	1.7
TMT-A	48 (20)	76 (40)	4.9ˆ*
TMT-B	84 (55)	167 (99)	5.4ˆ*
TMT-B-A	36 (49)	87 (75)	2.2
BNT	25.6 (3.2)	23.4 (3.9)	7.3ˆ**
FDS	8.3 (0.8)	8.2 (1.2)	0.1
BDS	5.4 (1.5)	4.4 (1.1)	5.3ˆ*
AVLT_Im	31.8 (5.6)	23.8 (6.3)	10.9ˆ***
AVLT_De	12.0 (2.3)	7.8 (3.1)	18.3ˆ***
AVLT_Clue	13.4 (1.7)	10.1 (2.8)	15.9ˆ***
AVLT_Re	13.8 (1.4)	11.9 (2.6)	4.0ˆ*
CDR-SB	0	1.4 (0.8)	48.1ˆ***
GDS	3.2 (3.1)	4.6 (2.6)	1.4
NPI	0.6 (1.9)	1.4 (1.4)	1.5

*For age and education, independent *t*-tests were performed. For gender and genotype, a chi-square test was performed (*df* = 1). For all other variables, one-way ANCOVA tests were performed by setting the group as an independent variable and age, education and gender as covariates (*df* = 1,55). ^∗^*p* < 0.05, ^∗∗^*p* < 0.01, ^∗∗∗^*p* < 0.001. M, male; F, female; HIS, Hachinski Ischemia Scale; Fazekas, Fazekas scale; MMSE, Minimum Mental State Examination; MoCA, Montreal Cognitive Assessment; CDT, clock drawing test; ADL, Activities of Daily Living scale; TMT, Trial Making Test; TMT-B-A, the difference between TMT-B and TMT-A; BNT, Boston Naming Test; FDS, forward digit span; BDS, backward digit span; AVLT, the WHO-UCLA Auditory Verbal Learning Test (Im, immediate; De, delay; Re, recognition); CDR-SB, Clinical Dementia Rating Scale Sum of Boxes; GDS, Geriatric Depression Scale; NPI, Neuropsychiatric Inventory.*

### NbM Volume

For cholinergic basal forebrain analysis, using previously developed stereotaxic cytoarchitectonic maps, the NbM volume versus intracranial volume ratio was comparable between the Control group (Mean ± SD, 0.0011 ± 0.00014) and the VCIND group (0.0011 ± 0.00011). The group differences did not reach statistical significance with or without controlling for age, gender and education (*Fs* < 1).

### Cholinergic Pathways

**FIGURE 2:**
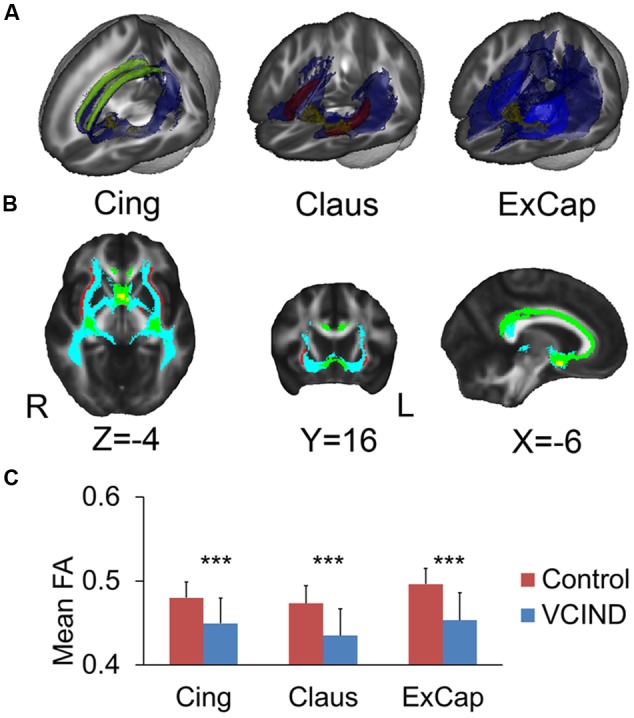
**Tracked pathways and FA values. (A)** 3D Reconstructed pathways (blue) for each division and the ROIs. Yellow for NbM, green for bilateral cingulum, light blue for external capsule and red for claustrum. **(B)** Overlapped pathways. Yellow for NbM seed, green for the pathway passing through the cingulum, light blue for the pathway passing through the external capsule, and red for the pathway passing through the claustrum. **(C)** Significantly higher FA values in the Control group compared with the VCIND group in all three cholinergic pathways. Cing, cingulum; ExCap, external capsule; Claus, claustrum. ^∗∗∗^*p* < 0.001.

**Figures 2A,B** demonstrates the tracked medial and lateral WM pathways in the probabilistic tractography analyses. The medial pathway from the NbM went through the genu portion of the corpus callosum and then targeted at the cingulum. For the lateral pathway, the ExCap and Claus divisions were both successfully tracked. The ExCap division projected to the inferior frontal cortex via the anterior corona radiata. The ExCap division also projected to the parietal and temporal cortex via the posterior thalamic radiation and superior longitudinal fasciculus. Additionally, the ExCap division targeted the splenium of corpus callosum and merged with the occipital cortex along with the inferior longitudinal fasciculus. In the Claus division, the fibers projected to fibers along with the claustrum and external capsule merged with the inferior fronto-occipital fasciculus.

### Diffusivity Indices Results

Significantly higher FA values were identified in the Control group than the VCIND group for all three pathways [**Figure 2C**, *F*(1,55) = 13.8, 19.6 and 21.6 for Cing, Claus and ExCap, respectively, *ps* < 0.001]. After controlling for age, gender, and education, the significant group differences prevailed in all diffusivity indices except for DA and MO in the Cing pathway (**Table 2**). Interestingly, the VCIND group exhibited higher DR and MD values, but also higher DA values compared with the controls.

**Table 2:** Diffusive indices [mean value (*SD*)] in Control and VCIND groups.

	Control	VCIND	*F*(1,55)
**DA**			
Cing	0.00129 (0.00003)	0.00132 (0.00006)	3.6
Claus	0.00127 (0.00003)	0.00132 (0.00006)	11.1ˆ**
ExCap	0.00126 (0.00003)	0.00131 (0.00005)	10.0ˆ**
**DR**			
Cing	0.00058 (0.00003)	0.00064 (0.00005)	13.2ˆ***
Claus	0.00058 (0.00003)	0.00066 (0.00006)	21.1ˆ***
ExCap	0.00054 (0.00003)	0.00062 (0.00007)	18.9ˆ***
**MD**			
Cing	0.00082 (0.00002)	0.00087 (0.00006)	10.6ˆ***
Claus	0.00081 (0.00002)	0.00088 (0.00006)	19.4ˆ***
ExCap	0.00078 (0.00002)	0.00085 (0.00006)	16.9ˆ***
**MO**			
Cing	0.52 (0.03)	0.50 (0.04)	3.5
Claus	0.13 (0.01)	0.12 (0.01)	6.2ˆ*
ExCap	0.55 (0.02)	0.52 (0.03)	7.3ˆ**

*^∗^*p* < 0.05, ^∗∗^*p* < 0.01, ^∗∗∗^*p* < 0.001.*

### Correlations between Cholinergic Pathways and Neuropsychological Scores

Partial correlation analyses controlling for age, gender, and education were used to study the relationship between WM pathways and neuropsychological scores. For general cognitive assessment, we found a significant negative correlation between CDR-SB and FA in the three pathways, showing that better FA was associated with a lower dementia rating. We also found significant positive correlations between FA in the three pathways and MoCA and MMSE, revealing that a higher FA was associated with better cognitive function. For specific cognitive domains, a higher FA in the three pathways was associated with better memory function as measured by AVLT, language function as measured by BNT, processing speed as measured by TMT-A, and executive function as measured by CDT and BDS (**Table 3**).

**Table 3:** Partial correlations between FA and neuropsychological scores.

	Cing	Claus	ExCap
CDR	-0.46ˆ*	-0.44ˆ*	-0.64ˆ***
MoCA	0.48ˆ***	0.49ˆ***	0.56ˆ***
MMSE	0.34ˆ*	0.34ˆ*	0.38ˆ**
CDT	0.30ˆ*	0.24ˆ*	0.38ˆ**
TMT-A	-0.24	-0.30ˆ*	-0.38ˆ*
TMT-B	-0.27	-0.25	-0.34ˆ*
BNT	0.21	0.26	0.33ˆ*
BDS	0.37ˆ*	0.39ˆ**	0.32ˆ*
AVLT_Im	0.36ˆ**	0.39ˆ**	0.46ˆ**
AVLT_De	0.41ˆ**	0.46ˆ***	0.54ˆ***
AVLT_Clue	0.31ˆ*	0.41ˆ**	0.44ˆ**
AVLT_Re	0.34ˆ*	0.38ˆ**	0.43ˆ**

*^∗^*p* < 0.05, ^∗∗^*p* < 0.01, ^∗∗∗^*p* < 0.001.*

### Mediation Results

To reduce potential statistical comparisons, only those variables that were significantly correlated with FA in all three tracts were included. Therefore, FA mediations on the associations between diagnostic group and MoCA, MMSE, CDT, BDS and AVLT were tested.

For MMSE, the group but not the FA effect was significant when both FA and group were entered into the regression model, revealing that FA did not mediate the group-MMSE association. For MoCA and AVLT, both FA and group effects were significant when both FA and group were entered into the regression model (*ps* < 0.05), revealing that the FA partly mediated the group-cognition relationship. For CDT and BDS, significant group-cognition was absent when FA was entered into the regression model, but the FA effects were significant or tended to reach significance, revealing full mediation of FA on the group-cognition association. Specifically, as shown in **Table 4**, after controlling for FA in Cing, Claus and ExCap pathways, group-related variance in CDT was attenuated by 83.77, 91.67, and 99.17%, respectively, along with no significant group effect in the full model. For BDS, as shown in **Table 4**, after controlling for FA in Cing, Claus and ExCap pathways, group-related variance in CDT was attenuated by 78.57, 85.71, and 78.57%, respectively.

**Table 4:** Mediation models testing the effects of group and FA on the Group-CDT and Group-BDS score relationship.

	R2	R2 change	*F*	Percentage attenuation	Beta
**FA mediation on Group-CDT**
Model 1					
Group	0.12		6.2ˆ***		0.347ˆ*
Model 2					
FA of Cing	0.16		8.43ˆ**		0.30#
Group	0.18	0.02	4.75ˆ*	83.33	0.17
FA of Claus	0.21		12.15ˆ***		0.41ˆ*
Group	0.22	0.01	6.11ˆ***	91.67	0.09
FA of ExCap	0.23		13.16ˆ***		0.45ˆ*
Group	0.23	0.001	6.48ˆ**	99.17	0.04
**FA mediation on Group-BDS**					
Model 1					
Group	0.14		7.35ˆ**		0.37ˆ**
Model 2					
FA of Cing	0.17		9.19ˆ**		0.29#
Group	0.20	0.03	5.58ˆ**	78.57	0.21
FA of Claus	0.18		10.35ˆ**		0.32ˆ*
Group	0.20	0.02	9.69ˆ**	85.71	0.18
FA of ExCap	0.14		7.22ˆ**		0.22
Group	0.17	0.03	4.45ˆ*	78.57	0.23

*#*p* < 0.10, ^∗^*p* < 0.05, ^∗∗^*p* < 0.01, ^∗∗∗^*p* < 0.001.*

## Discussion

In contrast to the consistent findings of NbM shrinking in Alzheimer’s disease (Teipel et al., 2011; Grothe et al., 2012), it is still under debate whether atrophy of NbM volume occurs in VCI. In the present study, using the recently developed stereotaxic cytoarchitectonic maps of NbM based on combined magnetic resonance imaging and histology of postmortem brains (Teipel et al., 2005; Grothe et al., 2010), we revealed that no significant NbM atrophy was observed in VCIND. Furthermore, the consistency between our findings in VCIND patients, which represent an early stage of VCI, and previous findings in postmortem studies, which represent the advanced stage of VCI, suggests that there is no significant NbM atrophy across the entire disease spectrum.

The specific and accurate measurements of WM lesions along the cholinergic pathway have remained undefined. So far most of the WM lesions in the cholinergic system were observed in postmortem brain studies (Tomimoto et al., 2005; Jung et al., 2012) or measured by visual counting of WM hyperintensities within cholinergic pathways (Behl et al., 2007; Kim et al., 2013). Such methods could not specifically measure the cholinergic pathway lesions. Therefore, the present study applied probabilistic tractography analysis to track the potential WM pathway mapping to the cholinergic pathways. The two major fiber tracks that map to the cholinergic pathway were both reconstructed *in vivo*. The tractography revealed fiber projections from the NbM to the cingulum via the genu portion of the corpus callosum in the medial pathway and to the ExCap and Claus divisions in the lateral pathway, following the cholinergic pathways previously identified by immunohistochemical procedures (Selden et al., 1998). Within the pathways, we found an impaired WM microstructure in the VCIND patients compared with controls. The FA and diffusive component alteration patterns in the patients suggested a loss/disruption of both axons (Teipel et al., 2014) and myelin (Madden et al., 2012) as well as increased brain water content (Sen and Basser, 2005) in VCIND, an early stage of VCI. It also suggests that diffusive alteration may be an early marker for VCI, as noted in AD (Gold et al., 2014).

Using CHIPS measurement, the associations of ischemic damage in cholinergic pathways with global cognitive impairment (Kim et al., 2012, 2013) and executive dysfunction (Swartz et al., 2003; Behl et al., 2007) have been reported. However, the underlying mechanism was still undetermined. Here, based on the *in vivo* reconstructed pathways carrying cholinergic input, partial correlation analyses revealed that FA was correlated with general cognitive assessments in CDR-SB, MoCA, MMSE, and with single cognitive domain assessments in memory, language and executive function. Furthermore, although FA-cognition associations were identified, it is unclear whether these associations were due to contribution from a third variable or whether the group-related cognitive differences were due to the group FA differences. Mediation analysis revealed that FA decline along the cholinergic pathways could fully explain the group-related differences in executive functions, especially the scores measured in the CDT and BDS, and partly explain group-related variance in global cognition (MoCA) and memory (AVLT). This suggests that WML in cholinergic pathways contribute to cognitive dysfunction in VCIND.

Our results provided better understanding of the characteristics of cognitive impairment in VCI patients. Executive dysfunction is the characteristic impairment in subcortical VCI (Jokinen et al., 2006), which, as indicated by our results, could be fully explained by damage in WM microstructure. As noted by Mesulam (2012), cholinergic pathways do not encode the content of experiences but may instead influence their inhibitory processing in executive functions. Moreover, as shown in **Figure 2**, the lateral pathway projects fibers into anterior corona radiata, an important pathway connecting the frontal and basal ganglia executive loop. Such a distribution pattern of the WM fiber track is also consistent with the fact that the damage in WM microstructure fully mediates executive dysfunction. For memory and global cognition, the FA in ExCap division significantly explained the group-related variation in MoCA, and the FA in the ExCap and Claus significantly explained the group-related variation in AVLT. The role of the lateral pathway lesion in memory was consistent with its fiber projection tractography, which projects into the temporal lobe and merges with medial temporal memory-related regions. The lateral pathway also has widespread projections to the frontal, temporal and occipital lobes, and therefore lesions in the lateral pathway would affect global cognition.

A potential limitation in the interpretation of our data is the cross-sectional nature of the current study. Longitudinal observations from early stage VCIND to dementia would help us to better understand the dynamic changes and temporal role of cholinergic lesions in the pathogenesis of VCI. Moreover, a previous study revealed that the ascending cholinergic axons of the nucleus basalis are mostly unmyelinated (Wainer and Mesulam, 1990). It is hard to distinguish unmyelinated from myelinated axons in DTI. Thus, in future studies, a combination of DTI with MRS, a technique to investigate neurochemical alterations, may help to uncover the unmyelinated fiber axons in the cholinergic pathway. Thirdly, as demonstrated by post-mortem studies, the co-occurrence of VCI and other neurodegenerative disorders in the elderly is very frequent (McAleese et al., 2016). We could not exclude the possibility that the VCIND patients included in our study might also suffer from other confounding preclinical cognitive disorders. A further cross-validation study with properly controlled diagnostic biomarkers for AD, Lewy body diseases etc. would be needed. Finally, it should be noted that the preserved volume could not exclude the possibility of neuron atrophy in NbM.

In summary, using probabilistic tractography analysis, our study tracked the two major pathways carrying cholinergic input. Targeting the early stage of VCI, we further revealed that the WM decline along the cholinergic pathways, but not the NbM, is significantly impaired in VCIND. The disrupted WM pathways could fully explain the executive dysfunction and partly mediate the memory and global cognition impairments in VICND. Our results provided better understanding of the cognitive profiles in VCI patients and suggested that MRI-based *in vivo* tracking of cholinergic pathways together with NbM measurement may become a valuable *in vivo* marker for evaluating the cholinergic system in VCI and other cognitive disorders, including AD. A further cross-validation study with properly controlled diagnostic biomarkers for possible confounding neurodegenerative disorders would be needed.

## Author Contributions

Conception and design of the study: QL, ZZ, ST, YT, and JJ; acquisition and analysis of data: QL, ZZ, ST, JY, YX, YT, and JJ; drafting the manuscript: QL, ZZ, ST, YT, and JJ. All authors were involved in the final manuscript revision.

## Conflict of Interest Statement

The authors declare that the research was conducted in the absence of any commercial or financial relationships that could be construed as a potential conflict of interest.

## References

[B1] Baron R. M. Kenny D. A. (1986). The moderator mediator variable distinction in social psychological research – Conceptual, strategic, and statistical considerations. *J. Pers. Soc. Psychol.* 51 1173–1182. 10.1037/0022-3514.51.6.1173 3806354

[B2] Beaulieu C. (2002). The basis of anisotropic water diffusion in the nervous system – a technical review. *NMR Biomed.* 15 435–455. 10.1002/Nbm.782 12489094

[B3] Behl P. Bocti C. Swartz R. H. Gao F. Sahlas D. J. Lanctot K. L. (2007). Strategic subcortical hyperintensities in cholinergic pathways and executive function decline in treated Alzheimer patients. *Arch. Neurol.* 64 266–272. 10.1001/archneur.64.2.266 17296844

[B4] Black S. Roman G. C. Geldmacher D. S. Salloway S. Hecker J. Burns A. (2003). Efficacy and tolerability of donepezil in vascular dementia: positive results of a 24-week, multicenter, international, randomized, placebo-controlled clinical trial. *Stroke* 34 2323–2330. 10.1161/01.STR.0000091396.95360.E1 12970516

[B5] Erkinjuntti T. Kurz A. Gauthier S. Bullock R. Lilienfeld S. Damaraju C. V. (2002). Efficacy of galantamine in probable vascular dementia and Alzheimer’s disease combined with cerebrovascular disease: a randomised trial. *Lancet* 359 1283–1290. 10.1016/S0140-6736(02)08267-3 11965273

[B6] Fazekas F. Chawluk J. B. Alavi A. Hurtig H. I. Zimmerman R. A. (1987). MR signal abnormalities at 1.5 T in Alzheimer’s dementia and normal aging. *AJR Am. J. Roentgenol.* 149 351–356. 10.2214/ajr.149.2.351 3496763

[B7] Feng G. Chen Q. Zhu Z. Wang S. (2016). Separate brain circuits support integrative and semantic priming in the human language system. *Cereb. Cortex* 26 3169–3182. 10.1093/cercor/bhv148 26209843

[B8] Folstein M. F. Folstein S. E. McHugh P. R. (1975). “Mini-mental state.” A practical method for grading the cognitive state of patients for the clinician. *J. Psychiatr. Res.* 12 189–198. 10.1016/0022-3956(75)90026-6 1202204

[B9] Gold B. T. Zhu Z. Brown C. A. Andersen A. H. LaDu M. J. Tai L. (2014). White matter integrity is associated with cerebrospinal fluid markers of Alzheimer’s disease in normal adults. *Neurobiol. Aging* 35 2263–2271. 10.1016/j.neurobiolaging.2014.04.030 24866404 PMC4087077

[B10] Grothe M. Heinsen H. Teipel S. J. (2012). Atrophy of the cholinergic Basal forebrain over the adult age range and in early stages of Alzheimer’s disease. *Biol. Psychiatry* 71 805–813. 10.1016/j.biopsych.2011.06.019 21816388 PMC3701122

[B11] Grothe M. Zaborszky L. Atienza M. Gil-Neciga E. Rodriguez-Romero R. Teipel S. J. (2010). Reduction of basal forebrain cholinergic system parallels cognitive impairment in patients at high risk of developing Alzheimer’s disease. *Cereb. Cortex* 20 1685–1695. 10.1093/cercor/bhp232 19889714 PMC2912653

[B12] Hanyu H. Shimizu S. Tanaka Y. Hirao K. Iwamoto T. Abe K. (2007). MR features of the substantia innominata and therapeutic implications in dementias. *Neurobiol. Aging* 28 548–554. 10.1016/j.neurobiolaging.2006.02.009 16569466

[B13] Hong J. H. Jang S. H. (2010). Neural pathway from nucleus basalis of Meynert passing through the cingulum in the human brain. *Brain Res.* 1346 190–194. 10.1016/j.brainres.2010.05.088 20570664

[B14] Hughes C. P. Berg L. Danziger W. L. Coben L. A. Martin R. L. (1982). A new clinical scale for the staging of dementia. *Br. J. Psychiatry* 140 566–572. 10.1192/bjp.140.6.566 7104545

[B15] Jokinen H. Kalska H. Mantyla R. Pohjasvaara T. Ylikoski R. Hietanen M. (2006). Cognitive profile of subcortical ischaemic vascular disease. *J. Neurol. Neurosurg. Psychiatry* 77 28–33. 10.1136/jnnp.2005.069120 16361588 PMC2117424

[B16] Jung S. Zarow C. Mack W. J. Zheng L. Vinters H. V. Ellis W. G. (2012). Preservation of neurons of the nucleus basalis in subcortical ischemic vascular disease. *Arch. Neurol.* 69 879–886. 10.1001/archneurol.2011.2874 22393167 PMC4184885

[B17] Kalaria R. N. Maestre G. E. Arizaga R. Friedland R. P. Galasko D. Hall K. (2008). Alzheimer’s disease and vascular dementia in developing countries: prevalence, management, and risk factors. *Lancet Neurol.* 7 812–826. 10.1016/S1474-4422(08)70169-8 18667359 PMC2860610

[B18] Keverne J. S. Low W. C. Ziabreva I. Court J. A. Oakley A. E. Kalaria R. N. (2007). Cholinergic neuronal deficits in CADASIL. *Stroke* 38 188–191. 10.1161/01.STR.0000251787.90695.05 17122431

[B19] Kilimann I. Grothe M. Heinsen H. Alho E. J. Grinberg L. Amaro E. (2014). Subregional basal forebrain atrophy in Alzheimer’s disease: a multicenter study. *J. Alzheimers Dis.* 40 687–700. 10.3233/JAD-132345 24503619 PMC4120953

[B20] Kim H. J. Moon W. J. Han S. H. (2013). Differential cholinergic pathway involvement in Alzheimer’s disease and subcortical ischemic vascular dementia. *J. Alzheimers Dis.* 35 129–136. 10.3233/JAD-122320 23364137

[B21] Kim S. H. Kang H. S. Kim H. J. Moon Y. Ryu H. J. Kim M. Y. (2012). The effect of ischemic cholinergic damage on cognition in patients with subcortical vascular cognitive impairment. *J. Geriatr. Psychiatry Neurol.* 25 122–127. 10.1177/0891988712445089 22689705

[B22] Madden D. J. Bennett I. J. Burzynska A. Potter G. G. Chen N. K. Song A. W. (2012). Diffusion tensor imaging of cerebral white matter integrity in cognitive aging. *Biochim. Biophys. Acta* 1822 386–400. 10.1016/j.bbadis.2011.08.003 21871957 PMC3241892

[B23] McAleese K. E. Alafuzoff I. Charidimou A. De Reuck J. Grinberg L. T. Hainsworth A. H. (2016). Post-mortem assessment in vascular dementia: advances and aspirations. *BMC Med.* 14:129 10.1186/s12916-016-0676-5 PMC5011905 27600683

[B24] Mesulam M. (2012). Cholinergic aspects of aging and Alzheimer’s disease. *Biol. Psychiatry* 71 760–761. 10.1016/j.biopsych.2012.02.025 22482884 PMC3712351

[B25] Mesulam M. Siddique T. Cohen B. (2003). Cholinergic denervation in a pure multi-infarct state: observations on CADASIL. *Neurology* 60 1183–1185. 10.1212/01.WNL.0000055927.22611.EB 12682331

[B26] Mesulam M. M. Geula C. (1988). Nucleus basalis (Ch4) and cortical cholinergic innervation in the human brain: observations based on the distribution of acetylcholinesterase and choline acetyltransferase. *J. Comp. Neurol.* 275 216–240. 10.1002/cne.902750205 3220975

[B27] Salthouse T. A. (1993). Speed mediation of adult age-differences in cognition. *Dev. Psychol.* 29 722–738. 10.1037//0012-1649.29.4.722

[B28] Scheltens P. Leys D. Barkhof F. Huglo D. Weinstein H. C. Vermersch P. (1992). Atrophy of medial temporal lobes on MRI in “probable” Alzheimer’s disease and normal ageing: diagnostic value and neuropsychological correlates. *J. Neurol. Neurosurg. Psychiatry* 55 967–972. 10.1136/jnnp.55.10.967 1431963 PMC1015202

[B29] Selden N. R. Gitelman D. R. Salamon-Murayama N. Parrish T. B. Mesulam M. M. (1998). Trajectories of cholinergic pathways within the cerebral hemispheres of the human brain. *Brain* 121(Pt 12) 2249–2257. 10.1093/brain/121.12.2249 9874478

[B30] Sen P. N. Basser P. J. (2005). A model for diffusion in white matter in the brain. *Biophys. J.* 89 2927–2938. 10.1529/biophysj.105.063016 16100258 PMC1366791

[B31] Smith S. M. Jenkinson M. Johansen-Berg H. Rueckert D. Nichols T. E. Mackay C. E. (2006). Tract-based spatial statistics: voxelwise analysis of multi-subject diffusion data. *Neuroimage* 31 1487–1505. 10.1016/j.neuroimage.2006.02.024 16624579

[B32] Sperling R. Mormino E. Johnson K. (2014). The evolution of preclinical Alzheimer’s disease: implications for prevention trials. *Neuron* 84 608–622. 10.1016/j.neuron.2014.10.038 25442939 PMC4285623

[B33] Swartz R. H. Sahlas D. J. Black S. E. (2003). Strategic involvement of cholinergic pathways and executive dysfunction: does location of white matter signal hyperintensities matter? *J. Stroke Cerebrovasc. Dis.* 12 29–36. 10.1053/jscd.2003.5 17903901

[B34] Teipel S. J. Flatz W. H. Heinsen H. Bokde A. L. Schoenberg S. O. Stockel S. (2005). Measurement of basal forebrain atrophy in Alzheimer’s disease using MRI. *Brain* 128(Pt 11) 2626–2644. 10.1093/brain/awh589 16014654

[B35] Teipel S. J. Grothe M. J. Filippi M. Fellgiebel A. Dyrba M. Frisoni G. B. (2014). Fractional anisotropy changes in Alzheimer’s disease depend on the underlying fiber tract architecture: a multiparametric DTI study using joint independent component analysis. *J. Alzheimers Dis.* 41 69–83. 10.3233/JAD-131829 24577476

[B36] Teipel S. J. Meindl T. Grinberg L. Grothe M. Cantero J. L. Reiser M. F. (2011). The cholinergic system in mild cognitive impairment and Alzheimer’s disease: an in vivo MRI and DTI study. *Hum. Brain Mapp.* 32 1349–1362. 10.1002/hbm.21111 20672311 PMC5899896

[B37] Tohgi H. Abe T. Kimura M. Saheki M. Takahashi S. (1996). Cerebrospinal fluid acetylcholine and choline in vascular dementia of Binswanger and multiple small infarct types as compared with Alzheimer-type dementia. *J. Neural Transm. (Vienna)* 103 1211–1220. 10.1007/BF01271206 9013408

[B38] Tomimoto H. Ohtani R. Shibata M. Nakamura N. Ihara M. (2005). Loss of cholinergic pathways in vascular dementia of the Binswanger type. *Dement. Geriatr. Cogn. Disord.* 19 282–288. 10.1159/000084553 15785029

[B39] Wainer B. Mesulam M.-M. (1990). “Ascending cholinergic pathways in the rat brain,” in *Brain Cholinergic Systems* eds Steriade M. Biesold D. (Oxford: Oxford University Press) 65–119.

[B40] Wallin A. Sjogren M. Blennow K. Davidsson P. (2003). Decreased cerebrospinal fluid acetylcholinesterase in patients with subcortical ischemic vascular dementia. *Dement. Geriatr. Cogn. Disord.* 16 200–207. 10.1159/000072803 14512714

[B41] Wilkinson D. Doody R. Helme R. Taubman K. Mintzer J. Kertesz A. (2003). Donepezil in vascular dementia: a randomized, placebo-controlled study. *Neurology* 61 479–486. 10.1212/01.WNL.0000078943.50032.FC 12939421

[B42] Winblad B. Palmer K. Kivipelto M. Jelic V. Fratiglioni L. Wahlund L. O. (2004). Mild cognitive impairment–beyond controversies, towards a consensus: report of the International Working Group on Mild Cognitive Impairment. *J. Intern. Med.* 256 240–246. 10.1111/j.1365-2796.2004.01380.x 15324367

[B43] Zhang M. Y. Katzman R. Salmon D. Jin H. Cai G. J. Wang Z. Y. (1990). The prevalence of dementia and Alzheimer’s disease in Shanghai, China: impact of age, gender, and education. *Ann. Neurol.* 27 428–437. 10.1002/ana.410270412 2353798

